# Supplementary material for: Proapoptotic Index Evaluation of Two Synthetic Peptides Derived from the Coneshell Californiconus californicus in Lung Cancer Cell Line H1299
Source: Mar Drugs. 2019 Dec 20;18(1):10. doi: 10.3390/md18010010 (PMC7024154; doi:10.3390/md18010010)
Supplement: Supplementary file 1 [file marinedrugs-18-00010-s001.pdf]

Table S1. Cal14.1a´s structures conformations after molecular dynamics.

Conformation	Number of structures	Existence time (ns)	
1	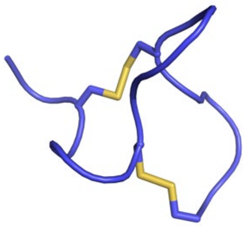	315	3.15
2	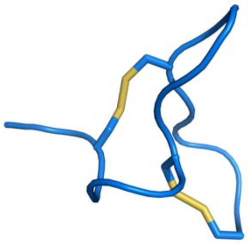	667	6.67
3	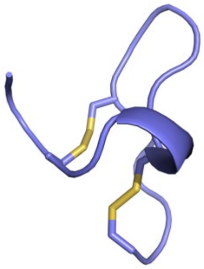	870	8.70
4	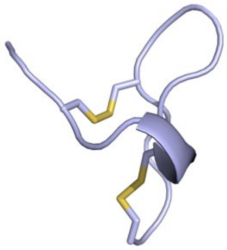	538	5.38
5	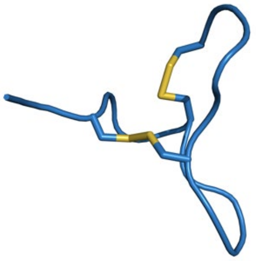	110	1.10

Table S2. Cal14.1b´s structures conformations after molecular dynamics.

Conformation	Number of structures	Existence time (ns)	
1	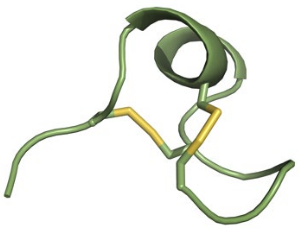	204	2.04
2	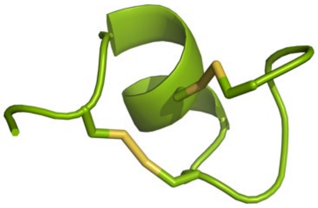	564	5.64
3	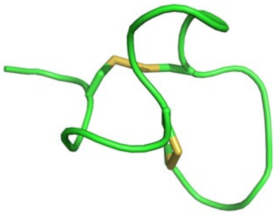	795	7.95
4	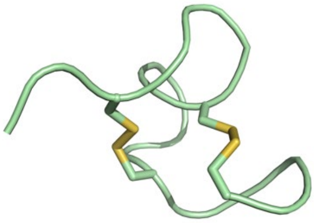	738	7.38
5	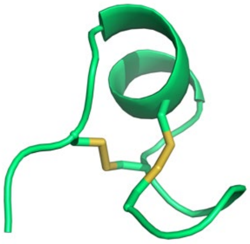	199	1.99
